# The Tyrosine Phosphatase SHP2 Associates with CUB Domain-Containing Protein-1 (CDCP1), Regulating Its Expression at the Cell Surface in a Phosphorylation-Dependent Manner

**DOI:** 10.1371/journal.pone.0123472

**Published:** 2015-04-13

**Authors:** Leslie Yewakon Gandji, Richard Proust, Lionel Larue, Franck Gesbert

**Affiliations:** 1 Institut Curie, Normal and Pathological Development of Melanocytes, Orsay, France; 2 Univ. Paris-Sud, Orsay, France; 3 CNRS, UMR3347, Bat 110, Orsay, France; 4 INSERM U1021, Bat 110, Orsay, France; 5 Equipe labellisée—Ligue Nationale contre le Cancer, Orsay, France; 6 INSERM UMR-S972, Hôpital Paul Brousse, Villejuif, France; Hungarian Academy of Sciences, HUNGARY

## Abstract

CUB domain-containing protein-1 (CDCP1) is a transmembrane glycoprotein that is phosphorylated by SRC family kinases (SFK) before recruiting and activating PKCδ. CDCP1 is overproduced in many cancers. It promotes metastasis and resistance to anoïkis. The robust production of CDCP1 would be associated with stemness and has been proposed as a novel prognosis marker. The natural transmembrane location of CDCP1 makes it an ideal therapeutic target and treatments based on the use of appropriate antibodies are currently being evaluated. However, we still know very little about the molecular fate of CDCP1 and its downstream signaling events. Improvements in our understanding of the molecular events occurring downstream of CDCP1 are required to make use of changes of CDCP1 production or functions for therapeutic purposes. By the mean of co-immunoprecipitation and affinity precipitation we show here, for the first time, that CDCP1 interacts directly, with the cytosolic tyrosine phosphatase SHP2. Point mutants of CDCP1 show that residues Y734 and Y743 are responsible for its interaction with SHP2. It may therefore compete with SFK. We also demonstrate that a shRNA-mediated down regulation of SHP2 is associated with a stronger CDCP1 phosphorylation and an impairment of antibody-mediated CDCP1 internalization.

## Introduction

CUB (complement protein subcomponents C1r/C1s, urchin embryonic growth factor and bone morphogenetic protein 1)-domain containing protein-1 (CDCP1) is an 836–amino acid, type 1 transmembrane glycoprotein. It has three potential CUB domains in its extracellular region and five phosphorylatable tyrosine residues in its intracytoplasmic part. It is overproduced in human colorectal cancer cells and in highly metastatic epidermoid carcinoma cell lines [1,2]. CDCP1 is a SRC-associated protein that is copurified with SRC and YES tyrosine kinases in MDA-468 breast cancer cells [3].

CDCP1 expression has been reported in several cancers, including tumors of the colon, prostate, kidney, lung and pancreas in particular, and in melanomas [2,4–7]. It is correlated with an increased resistance to anoikis, a typical apoptotic mechanism involving a loss of interaction between the cell and the substratum [5]. CDCP1 has been shown to be expressed in various cells with stemness profiles [8,9]. However, despite the growing number of publications on this subject, the function of CDCP1 remains a matter of debate. It has been suggested that CDCP1 acts as an oncogene, or, on the contrary, as a tumor suppressor [10,11]. The reasons for these apparent discrepancies between studies remain unclear and further investigations are required. CDCP1 has recently been proposed as a novel stem cell marker and as a diagnosis and prognosis marker for various cancers [8,12,13]. It may also constitute an interesting new treatment target, and it is thus becoming urgent to decipher the signaling molecules associated with CDCP1 and potentially contributing to its function in cancer progression.

Increases in CDCP1 levels have been shown to be accompanied by increases in SRC activity. As a consequence, CDCP1 is phosphorylated by SRC on its Y734 residue, facilitating direct associations between SRC and CDCP1 [5]. Thus, SRC reinforces CDCP1 phosphorylation, by directly phosphorylating the CDCP1-Y762 residue, which acts as the docking site for PKCδ [14]. A phosphoproteomic analysis of melanoma tumor cells and cell lines showed that metastatic cells expressed larger amounts of CDCP1 and that the forced expression of CDCP1 in melanoma cell lines led to the activation of SRC and to an increase in metastatic potential [7]. Furthermore, the use of a specific anti-CDCP1 antibody has been reported to induce the phosphorylation of CDCP1 and major signaling molecules, including GRB2, SHC and SHP2, accompanied by increased migratory properties of the cells [15].

The cytosolic tyrosine phosphatase SHP2 is the ubiquitously expressed product of the *PTPN11* gene. Multiple mutations of *PTPN11* have been shown to corrupt the functions of SHP2. These alterations, leading to the production of gain- or loss-of-function mutant forms of SHP2, are responsible for the Noonan or LEOPARD syndromes, respectively [16,17]. SHP2 is essential for the cell transformation process mediated by v-SRC and was the first cytosolic phosphatase to be identified as a *bona fide* oncogene [18,19]. SHP2 has two N-terminal SH2 domains, followed by a protein tyrosine phosphatase catalytic domain (PTP domain) [20]. Upon activation by growth factors or cytokine, SHP2 interacts, via its SH2 domains, with various partners containing phosphorylated tyrosine residues in a specific environment [21]. This interaction induces a change in conformation, disrupting the interaction of the N-SH2 domain with the PTP domain, leading to an activated state [22–24]. SHP2 transduces mitogenic, pro-survival and migratory signals from various receptors and it has been reported to regulate cell migration, focal adhesion dynamics and RHOA activity [17,25]. It may therefore play an important role in the triggering of metastasis [26]. Much effort has been devoted to the identification of molecules interacting with or serving as substrates for SHP2, but very few have been described to date.

The tyrosine phosphorylation of CDCP1 has been reported to play a crucial role in oncogenesis. However, the signals regulating the phosphorylation of CDCP1 and the fate of this protein at the cell surface are largely unknown. As the functions of CDCP1 and SHP2 seem to overlap, we investigated whether these two proteins interacted and whether SHP2 could regulate CDCP1 functions. We show here that SHP2 interacts directly, in a tyrosine phosphorylation-dependent manner, with both the Y734 and Y743 tyrosine residues of CDCP1. We also show that CDCP1 phosphorylation is greatly increased in the absence of SHP2. Furthermore, we demonstrate that SHP2 is required for the regulation of CDCP1 expression at the cell surface.

## Materials and Methods

### Antibodies and reagents

Monoclonal antibodies directed against SHP2, pTyr(4G10), and c-myc(9E10) were purchased from BD Biosciences (Le Pont de Claix, France), Upstate Biotechnology (Millipore, Molsheim, France) and Santa Cruz Biotechnology (Heidelberg, Germany), respectively. Polyclonal antibodies directed against p-CDCP1 (pY734) were purchased from Cell Signaling Technology (Ozyme, Saint-Quentin-en-Yvelines, France). The monoclonal antibody against GST (15TF-1D10) and the polyclonal antibody against CDCP1 were obtained from Neo Biotech (CliniSciences, Nanterre, France) and Pierce Biotechnology (Thermo Fischer Scientific, Courtaboeuf, France), respectively. The monoclonal anti-HA antibody was from Abcam (HA-11). The anti-tubulin antibody (TUB 2.1) was from Sigma (Sigma-Aldrich, Lyon, France).The monoclonal antibody against CDCP1 (CUB1) was obtained from Biolegend (Ozyme, Saint-Quentin en Yvelines, France).

### Plasmids and site-directed mutagenesis

SHP2 and CDCP1 expression constructs were kind gifts from B.J. Neel (Ontario Institute for Cancer Research, Toronto, Canada) and J. Bréard (INSERM U-1004, Hopital Paul Brousse, France), respectively. The Y734F and Y743F CDCP1 mutations were generated by site-directed mutagenesis (QuikChange II XL Site-Directed Mutagenesis; Stratagene, Agilent Technologies, Les Ulis, France), according to the kit manufacturer’s instructions. The following primers were used (the substituted nucleotides are shown in bold and underlined):

- CDCP1-Y734F-forward: 5'-GACTCCCATGTGT**T**TGCAGTCATCGAG-3'
- CDCP1-Y734F-reverse: 5'-CTGGATGACTGCA**A**ACACATGGGAGTC-3'
- CDCP1-Y743F-forward:5'-GGACACCATGGTAT**T**TGGGCATCTGCTAC-3'
- CDCP1-Y743F-reverse: 5'-GTAGCAGATGCCCA**A**ATACCATGGTGTCC-3'

Mutations were verified by DNA sequencing (MWG-Eurofins).

Two shRNA, D1 and D2, directed against SHP2 were used in this study and were obtained from Open Biosystem/Dharmacon. shD1, ref # TRCN0000005003 targets the sequence 5’-CGCTAAGAGAACTTAAACTTT-3’ and shD2, ref # TRCN0000005004 targets the sequence 5’-GCTGAAATAGAAAGCAGAGTT-3’ of the human SHP2 sequence (Human PTPN11, NM_002834).

The GST-SHP2-SH2 construct has been described elsewhere [27]. The GST-DACS mutant was generated by site-directed mutagenesis converting D425 to A425 and C459 to S459, as previously described by others [28].

### Cell lines, transfections and treatments

HeLa cells (ATCC, CCL-2) and colorectal carcinoma HCT 116 cells (ATCC, CCL-247) were grown at 37°C in Dulbecco's Modified Eagle Medium (DMEM) supplemented with 10% fetal bovine serum, 1% penicillin-streptomycin and 1mM sodium pyruvate, under an atmosphere containing 5% CO_2_. HeLa cells expressing CDCP1, shSHP2-D1 and shSHP2-D2 were obtained by transfection in the presence of Fugene-6 transfection reagent (Promega, Charbonnières, France), according to the manufacturer’s instructions. Cell lines displaying stable expression were obtained by selection in complete medium supplemented with blasticidin or puromycin, depending on the selection cassettes present in the constructs. The concentration of the selection agents was initially adjusted for each cell line. CDCP1-positive cells were sorted with a FACS-Vantage SE cell sorter (Beckton Dickinson, Le Pont de Claix, France). Cells were treated with 25 μM pervanadate (PerVO_3_) or with 5μg/ml anti-CDCP1 antibodies for 15 minutes at 37°C, rinsed in cold PBS 1X and lysed as described below.

### Cell lysis, immunoprecipitation and GST pull-down assays

Cells were lysed in 1% Brij O10 lysis buffer (50mM Tris pH8, 150mM NaCl, 10mM NaF, 1mM EDTA pH8, 1mM EGTA, 1% Brij O10) supplemented with 1mM PMSF, 1mM vanadate, 1/100 protease inhibitor cocktail (Sigma-Aldrich, Saint Quentin Fallavier, France). Lysates were clarified by centrifugation at 17,000 x g, for 20 minutes at 4°C. In immunoprecipitation and GST-pull-down assays, equivalent amounts of protein were incubated with the specified antibody or fusion protein for 2 h at 4°C. Complexes were immobilized on G protein- or glutathione-coupled beads, washed in lysis buffer supplemented with 1mM vanadate and boiled for 5 minutes in Laemmli's buffer.

### Western blot and overlay assays

Proteins were resolved by SDS-PAGE and transferred onto a PVDF membrane. The membrane was then blocked by incubation in 0.2% Tween 20 in TBS (TBS-T) and 5% BSA, and incubated with the appropriate primary antibody. Secondary antibodies (Alexa Fluor 680-conjugated goat anti-mouse or goat anti-rabbit antibodies, Life Technologies, Saint-Aubin, France) were incubated with the membrane, which was then washed with TBS-T. In overlay assays, the membrane was first incubated for 1h with the fusion protein and then processed as in western blotting experiments. Proteins were visualized with the Odyssey Infrared Detection System (LI-COR Biosciences, Eurosep Instruments, Cergy-Pontoise, France).

### CDCP1 internalization assay

Cells were detached and incubated for 1h at 4°C with anti-CDCP1 antibody diluted 1/200 in complete medium. Cells were washed three times (by resuspension in complete medium and centrifugation at 800 x *g* for 3 minutes) and then incubated at 37°C for the times indicated, for internalization of the CDCP1-primary antibody immunocomplex. The cells were then incubated for 1h at 4°C with Alexa Fluor 647-conjugated donkey anti-mouse antibody (Abcam, Cambridge, UK) and washed 3 times. All samples were then acquired with a FACSCalibur machine (Beckton Dickinson, Le Pont de Claix, France) and the results obtained were analyzed with FlowJo software (Tree Star).

## Results

### The intracytoplasmic domain of CDCP1 contains an ITAM-like structure and allows its co-immunoprecipitation with SHP2

**Fig 1:**
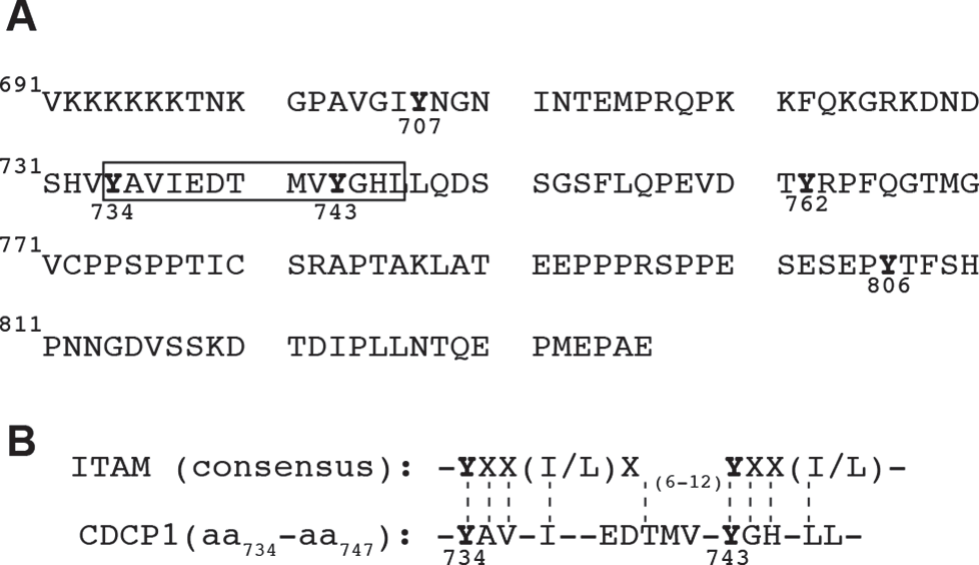
Primary sequence of intracellular CDCP1. **A.** Amino acids are numbered from the first intracellular residue. Phosphorylatable tyrosine residues are shown in bold typeface and are numbered. The ITAM-like motif is shown in a box. **B.** Alignment of the ITAM-like motif of CDCP1 with the consensus sequence of an ITAM motif. Phosphorylatable residues are shown in bold typeface and are numbered according to the corresponding CDCP1 sequence.

CDCP1 associates with SRC family kinases and PKCδ [5,14]. The Y734 tyrosine residue is the principal site of tyrosine phosphorylation by SFK in CDCP1 [29,30]. An *in silico* analysis of the primary sequence of CDCP1 revealed that the tyrosine residues Y734 and Y743 were both located in an YXXI/L consensus sequence (YAVI and YGHL respectively, Fig 1A). As aligned in Fig 1B, the overall sequence surrounding residues Y734 and Y743 is very close to an Immunoreceptor Tyrosine-Based Activation Motif (ITAM) that could act as a docking site for the tyrosine phosphatase SHP2.

**Fig 2:**
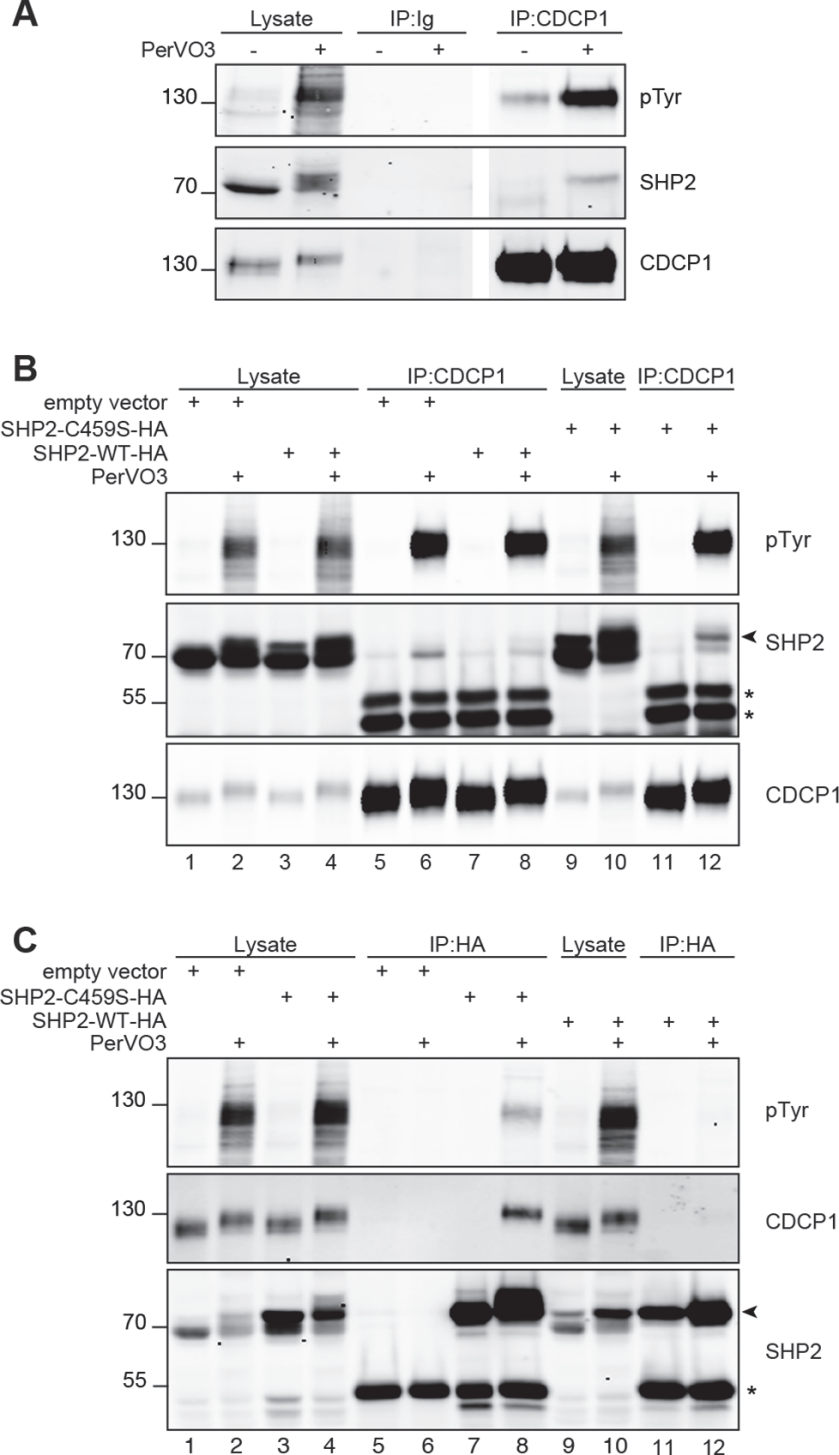
Co-immunoprecipitation of SHP2 and CDCP1. **A.** PC3 cells were left untreated or were treated for 15 minutes with 25μM pervanadate (PerVO_3_). The cells were lysed and the lysates were subjected to immunoprecipitation (IP) with anti-CDCP1 control antibodies, as indicated. Total cell lysates and immunoprecipitates were analyzed by western blotting with the antibodies indicated. **B and C.** HCT 116 cells were transfected with an empty vector or wild-type SHP2 (SHP2-WT-HA) or dominant-negative SHP2 mutant (SHP2-C459S-HA) expression constructs, as indicated. Forty-eight hours after transfection, the cells were left untreated or were treated for 15 minutes with 25μM pervanadate (PerVO_3_). The cells were lysed and the lysates were subjected to immunoprecipitation (IP) with anti-CDCP1 (A) or anti-HA (B) antibodies, as indicated. Total cell lysates and immunoprecipitates were analyzed by western blotting with the antibodies indicated. The position of the exogenously expressed SHP2 constructs that migrated more slowly, due to a fused HA-Tag, are indicated by an arrowhead. The immunoglobulin heavy chains (IgH) are indicated by asterisks (*). The results shown are representative of at least four independent experiments.

The PC-3 pancreatic cancer cell line was shown to express high levels of CDCP1 and SHP2. A series of co-immunoprecipitation experiments revealed the presence of an SHP2-CDCP1 complex in this cell lines treated for 15 minutes with pervanadate (PerVO_3_), a protein tyrosine phosphatase inhibitor that increases the level of tyrosine phosphorylation (Fig 2A).

We further confirmed these results in the HCT 116 colorectal cancer cell line, which expresses both SHP2 and CDCP1 (Fig 2B and 2C, lane 1 middle panels). HCT 116 cells were transfected with either an empty vector, or with expressing vectors encoding the WT or the C459S mutant of SHP2 as indicated (Fig 2). The C459S “substrate trapping” mutant construct encodes a mutant form of SHP2 devoid of catalytic activity that remains associated with its substrate [31–33]. Before lysis, 48h after transfection, the cells were either left untreated or were treated with PerVO_3_. Confirming our previous findings, the endogenous SHP2 was co-immuno-precipitated with CDCP1 when the cells were treated with PerVO_3_ (Fig 2B middle panel, lanes 5 and 6). This co-immunoprecipitation was confirmed in cells transfected with the WT SHP2 construct. Furthermore, co-immunoprecipitation was more efficient following the transfection of cells with the SHP2-C459S mutant construct (Fig 2B, lanes 11 and 12). In our hands, the commercially available anti-SHP2 antibodies did not provide any convincing co-immunoprecipitation. We turned to transfection and immunoprecipitation of an HA tagged SHP2 with the anti-HA antibody. The SHP2 expression constructs ran as higher molecular species as they bear a C-terminal HA tag. In this experiment, as expected given that only the HA-tagged species could be precipitated, CDCP1 was not precipitated from mock-transfected cells (Fig 2C middle panel, lanes 5 and 6). A faint band corresponding to CDCP1 was barely detectable in PerVO_3_-treated cells transfected with the SHP2-WT construct (Fig 2C middle panel, lane 12). However, clear co-immunoprecipitation was observed in PerVO_3_-treated cells transfected with the catalytically inactive SHP2-C459S construct (Fig 2C, lane 8). CDCP1 migrated more slowly following the treatment of the cells with PerVO_3_ (Fig 2B and 2C, lower and middle panels, respectively), probably due to the phosphorylation status of CDCP1. Furthermore, CDCP1 was phosphorylated exclusively in PerVO_3_-treated cells. Our results indicate that SHP2 and CDCP1 interact in a phosphorylation-dependent manner.

**Fig 3:**
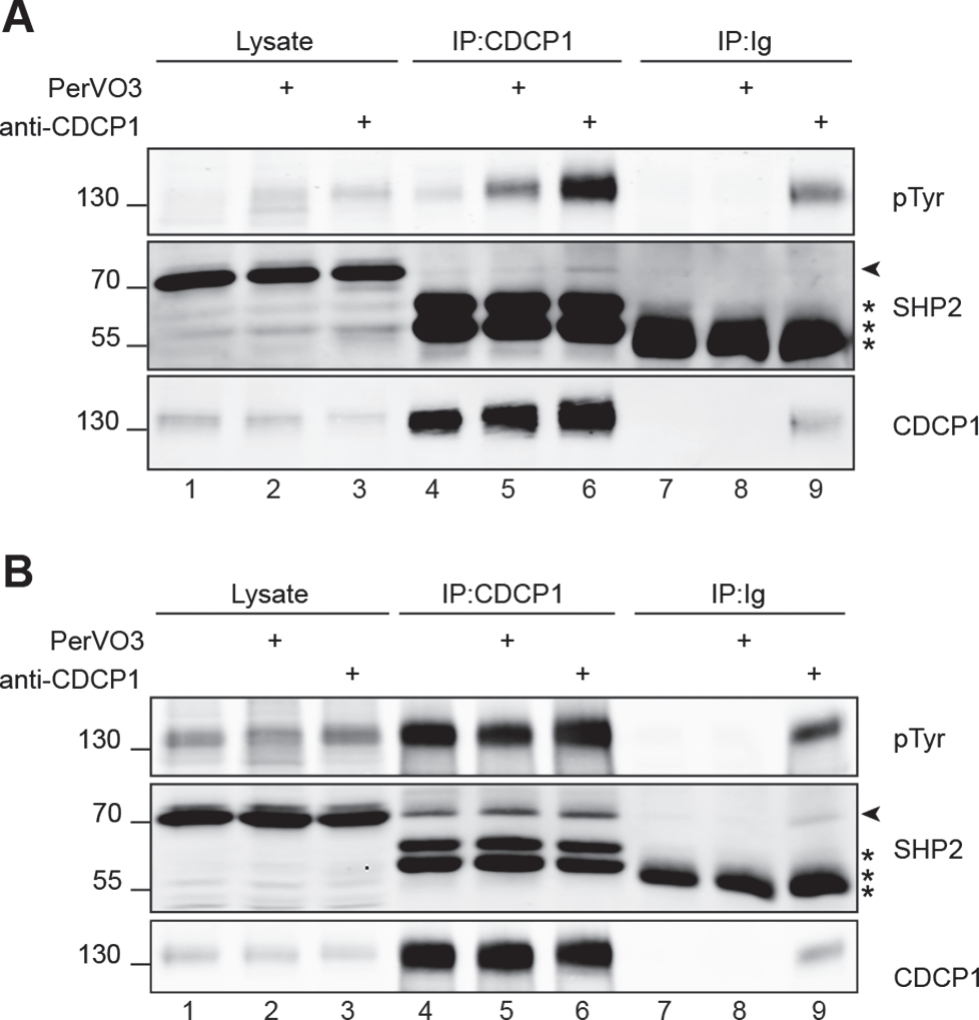
Endogenous SHP2 and CDCP1 co-immunoprecipitate CDCP1 triggered HCT 116 cells. HCT 116 cells were plated at **(A)** 0.2x10^6^ cells/ml (non confluent) or **(B)** 1.4x10^6^ cells/ml (confluent) for 48h. The cells were left untreated or were incubated with 25μM PerVO3 or 5μg/ml anti-CDCP1 antibody, as indicated, for 15 minutes. The cells were lysed and anti-CDCP1 antibodies were used for immunoprecipitation (IP), as indicated and as described in the experimental procedures section. Control immunoprecipitations were performed with irrelevant mouse immunoglobulins (Ig). Total cell lysates and immunoprecipitates were analyzed by western blotting with anti-pTyr, anti-CDCP1 and anti-SHP2 antibodies. The results shown are representative of at least three independent experiments. The position of endogenous SHP2 is indicated by an arrowhead. The asterisks (*) indicate the position of the Ig Heavy chains used for the immunoprecipitation or the triggering of CDCP1.

The tyrosine phosphorylation status of CDCP1 in HCT 116 cells is affected by the degree of confluence of the cells in culture [15]. In addition, it has been described that some antibodies directed against the extracellular domain of CDCP1 can induce, in culture, the tyrosine- phosphorylation of CDCP1 and the induction of signaling events, just as it would be expected from a ligand [15]. HCT 116 cells were maintained non-confluent in culture. The cells were either left untreated or were incubated 15 minutes with PerVO3 or with 5μg/ml Cub1 anti-CDCP1 antibody. We observe that in these conditions CDCP1 is very weakly phosphorylated in non-treated cells (Fig 3A, upper panel, lane 4). When the non-confluent cells were incubated in the presence of an anti-CDCP1 antibody, we could confirm that the phosphorylation of CDCP1 was greatly increased, even more than in PerVO3-treated cells (Fig 3A, upper panel, compare lanes 6 and 5, respectively). The control IPs with a non-relevant antibody show the specificity of the immunoprecipitations (Fig 3A, IP:Ig). It is not surprising to observe the presence of CDCP1 in the control IP in the anti-CDCP1 treated cells as the CDCP1-bound antibody is precipitated. In non-treated and PerVO3 treated cells, we could observe that the precipitation of SHP2 with CDCP1 is hardly detectable (Fig 3A, middle panel, lanes 4 and 5). However, we could detect the presence of SHP2 when the cells were triggered with an anti-CDCP1 (Fig 3A, middle panel, lane 6).

HCT 116 cells were maintained at full confluence for 48h and the cells were left untreated or incubated with PerVO3 or with an anti-CDCP1 as described above. In these conditions, we observed that the basal level of phosphorylation of CDCP1 was already maximal and could not be increased by our treatments (Fig 3B, upper panel, lanes 4, 5 and 6). In these conditions, the interaction of endogenous CDCP1 and SHP2 was clearly visible (Fig 3B, middle panel, lanes 4, 5 and 6). In addition, and as explained above, we could also precipitate CDCP1 with its triggering antibody in the control IPs (Fig 3B, lane 9). In this condition we could effectively co-immuno-precipitate SHP2 (Fig 3B, middle panel, lane 9).

When comparing Fig 3A and 3B lane 4 we show that CDCP1 phosphorylation and its association with SHP2 can be modulated through the only cell confluence parameter.

Altogether, our data identify SHP2 as a novel interactor of CDCP1. The interaction of SHP2 with CDCP1 appears to be dependent on the tyrosine phosphorylation of CDCP1. In our conditions, the phosphorylation of CDCP1, and its association with SHP2, can be modulated by various stimuli such as addition of PerVO3 or cell confluence or through an antibody-mediated triggering of CDCP1 in a “ligand-like” manner as described by others [15,34,35].

### SHP2 interacts only with the phosphorylated form of CDCP1 via its SH2 domains

**Fig 4:**
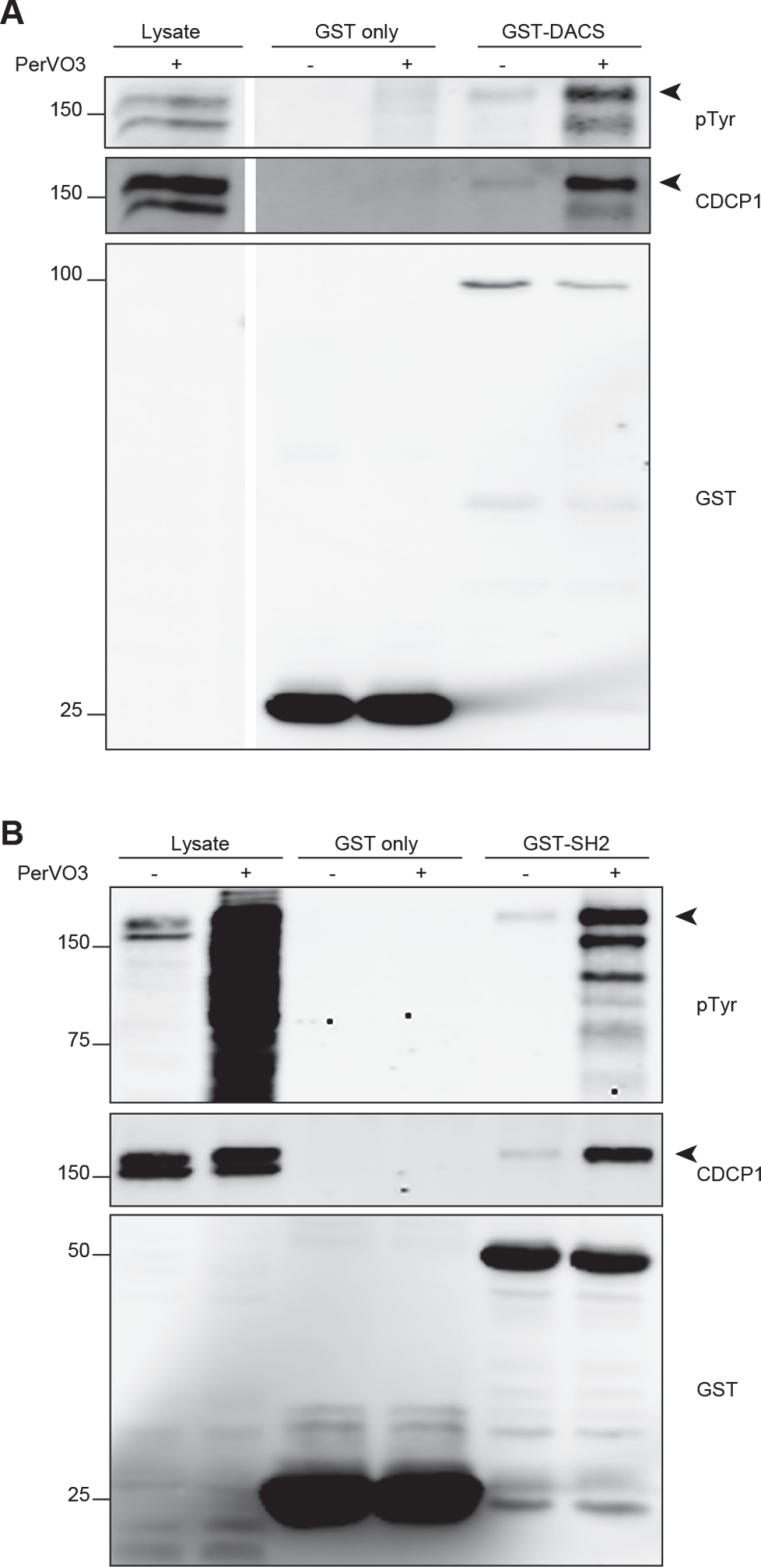
Phosphorylated CDCP1 is efficiently pulled down by recombinant SHP2 substrate trapping mutant or SHP2-SH2 domains. HeLa cells stably expressing CDCP1 were left untreated or were treated for 15 minutes with 25μM pervanadate (PerVO_3_), as indicated. Cell lysates were subjected to GST-pull down assays with 5 μg of GST protein alone (GST only), GST fused to a SHP2 substrate trapping mutant (GST-DACS) or GST fused to the SHP2-SH2 domains (GST-SH2), as mentioned in A and B. The affinity-purified complexes were resolved by SDS-PAGE and analyzed by immunoblotting with the indicated antibodies. In some conditions, particularly in HeLa cells, CDCP1 was detected as two species, the more slowly migrating species being tyrosine-phosphorylated (Fig 4A and 4B, compare lower and middle panels, the arrowhead indicates the slower migrating species). The data shown are representative of more than eight independent experiments.

GST pull-down experiments were performed to confirm that CDCP1 interacted with a DACS “substrate trapping” mutant of SHP2 devoid of catalytic activity that remains bound to its substrate [28] (Fig 4A) or with the SH2 domains of SHP2 (Fig 4B). We stably transfected HeLa cells, which do not naturally express CDCP1, with a construct encoding CDCP1 (HeLa-CDCP1 cells). The cells were treated or not with PerVO_3_, as indicated. With both GST constructs, we observed the efficient pull-down of a major tyrosine-phosphorylated protein species with a molecular weight of about 150 kDa (Fig 4A and 4B, pTyr upper panels), which we confirmed to be CDCP1 (Fig 4A and 4B, middle panels). This interaction was greatly enhanced by PerVO_3_ treatment.

Our data provide the first evidence for a tyrosine phosphorylation-dependent interaction between CDCP1 and SHP2. We also show that this interaction is most probably mediated through the SHP2-SH2 domains with a tyrosine-phosphorylated population of CDCP1.

### SHP2 interacts directly with the phosphorylated Y734 and Y743 residues of CDCP1

CDCP1 and SHP2 are part of a common molecular complex. We therefore determined whether this interaction was direct. Our data also strongly suggested that the interaction between SHP2 and CDCP1 was mediated by the SHP2-SH2 domains. We therefore tried to identify the precise residues of the CDCP1 intracytosolic region involved in this interaction. We used overlay assays to demonstrate direct interactions, as this method has been shown to be both specific and straightforward.

**Fig 5:**
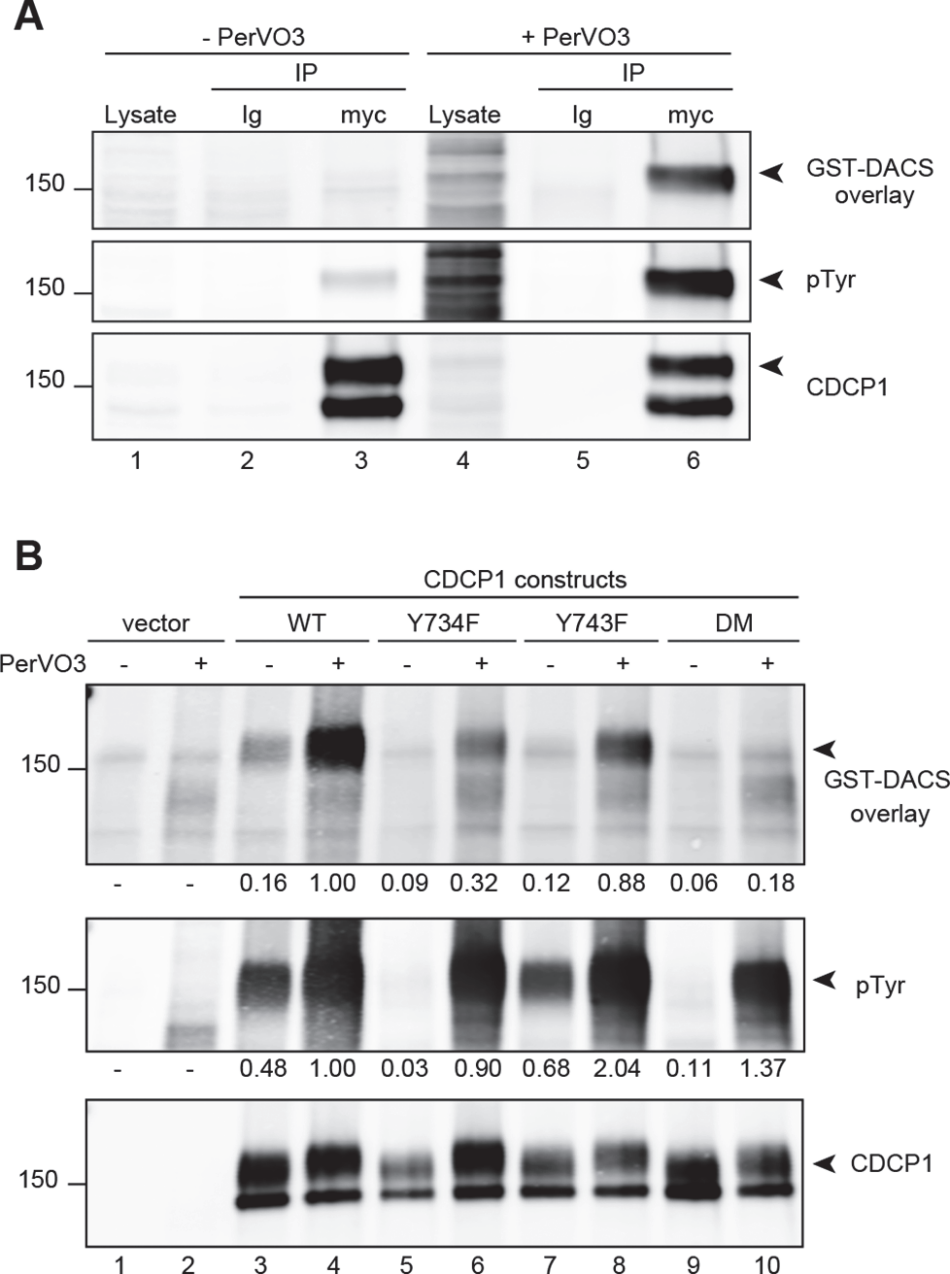
SHP2 interacts directly with the phosphorylated tyrosine residues in positions 734 and 743 of CDCP1. **A.** HeLa cells were transfected with a CDCP1-myc expression construct and were left untreated or were treated, 48h after transfection, with 25μM pervanadate (PerVO_3_). The cells were lysed and CDCP1 was immunoprecipitated with an anti-Myc antibody (myc). Mouse immunoglobulins were used as a negative control, as indicated. The immune complexes were resolved by SDS-PAGE and transferred on membranes. An overlay assay was performed, as described in the experimental procedures, with a purified SHP2 trapping mutant protein fused to GST (GST-DACS, upper panel). The membrane was then stripped and reprobed with antibodies against phosphorylated tyrosine residues (middle panel) and CDCP1 (bottom panel). In HeLa cells, CDCP1 was detected as a doublet, only the upper band of which (arrowhead) was found to be phosphorylated and to interact with SHP2. **B.** HeLa cells were transfected with a construct encoding the wild-type CDCP1 (WT), or a CDCP1 protein with substitution of the Y734 or Y743 tyrosine residues (Y734F or Y743F, respectively) or both (DM). All these constructs were Myc-tagged. The cells were treated and lysed as described above. The intensities of the signals were analyzed and were normalized on the signal obtained for the CDCP1 blot (lower panel). The normalized value is considered to be 100% in CDCP1-WT transfected cells treated with PerVO_3_ (lane 3). The values are expressed as fold increase or decrease. The data shown are representative of at least five independent experiments.

Lysates from untreated or PerVO_3_-treated HeLa-CDCP1 cells were first subjected to immunoprecipitation with a control immunoglobulin or with an anti-myc antibody (Fig 5A, lanes 2 and 5, and 3 and 6, respectively). The resolved immunoprecipitates were transferred onto PVDF membranes, which were incubated with a purified solution of the GST-DACS protein. The membranes were incubated with the appropriate antibodies for the detection of bound GST-DACS, demonstrating direct interaction of the recombinant SHP2 protein with phosphorylated CDCP1 from PerVO_3_-treated cells (Fig 5A, upper panel, lane 6). As a control, we used lysates subjected to immunoprecipitation with an irrelevant immunoglobulin (Fig 5A, lanes 2 and 5) or lysates of untreated cells; no interaction was detected in the controls (Fig 5A, lane 3).

As the interaction of SHP2 with CDCP1 was found to be direct, we hypothesized that it would involve one or both of the tyrosine residues (Y734 and/or Y743) of CDCP1, located in the previously described ITAM-like structure. We generated point mutant constructs for CDCP1, encoding proteins in which one of the tyrosine residues was replaced with a phenylalanine residue (CDCP1-Y734F and-Y743F mutants) or both tyrosine residues were simultaneously replaced (CDCP1-DM double mutant). HeLa cells were transiently transfected with an empty vector or with the WT or mutants constructs of CDCP1, as indicated. Forty-eight hours after transfection, the cells were left untreated or were treated with PerVO_3_ before the precipitation of CDCP1 with an antibody binding to its Myc-Tag.

Interestingly, after PerVO_3_ treatment, the levels of tyrosine phosphorylation of the CDCP1 constructs were similar (Fig 5B, middle panel, lanes 4,6,8 and 10). However, the CDCP1-Y734F and-DM proteins were not phosphorylated in the absence of PerVO_3_, by contrast to the CDCP1-WT and-Y743F forms (Fig 5B, middle panel, compare lanes 5 and 9 to lanes 3 and 7, respectively). In the presence and absence of PerVO_3_, CDCP1-Y743F had a phosphorylation profile similar to that of the WT construct (Fig 5B, middle panel, lanes 3 and 7 and 4 and 8). These results confirm that CDCP1 can be phosphorylated on several tyrosine residues other than Y734 and Y743, such as Y762. Moreover, our data indicate that CDCP1 can still be phosphorylated even in the absence of the key Y734 residue.

As in Fig 5A, the CDCP1 constructs were immunoprecipitated and used for overlay assays with GST-DACS (Fig 5B). These assays confirmed the direct interaction of SHP2 with the phosphorylated form of CDCP1, and we observed a large increase of the interaction after PerVO_3_ treatment, this increase being correlated with the increase in CDCP1 phosphorylation (Fig 5B, upper panel lanes 3 and 4). This direct interaction was much weaker for the Y734F mutant, although CDCP1 was still strongly phosphorylated (Fig 5B, upper panel, compare lanes 4,6 and 8). We could observe that the level of phosphorylation of the Y734F mutant, after a PerVO3 treatment, was only decreased by 10% whereas the interaction with SHP2 was decreased by 70%. On the other hand, the phosphorylation of the Y743F mutant appeared to increase whereas the interaction with SHP2 decreased by 12%. Furthermore, the direct interaction between CDCP1 and SHP2 was dramatically diminished by mutations converting both Y734 and Y743 into F residues (Fig 5B, upper panel, lanes 9 and 10). In conclusion, we found that CDCP1 was still phosphorylated if Y734 or/and Y743 residues are mutated. This latter observation supports the idea that our Far Western conditions are specific and that the soluble SH2 domain does not randomly interact with any phosphorylated residue. These results confirm our hypothesis that SHP2 interacts directly with both the Y734 and Y743 residues, which are located in an ITAM-like structure in the intracellular region of CDCP1.

### SHP2 regulates the level of phosphorylation of CDCP1

As SHP2 interacted directly with CDCP1 (Fig 5) and this interaction was found to be stronger in the presence of a catalytically inactive “substrate trapping” C459S mutant of SHP2 (Fig 2), we investigated whether SHP2 could regulate the level of CDCP1 phosphorylation through its phosphatase activity.

**Fig 6:**
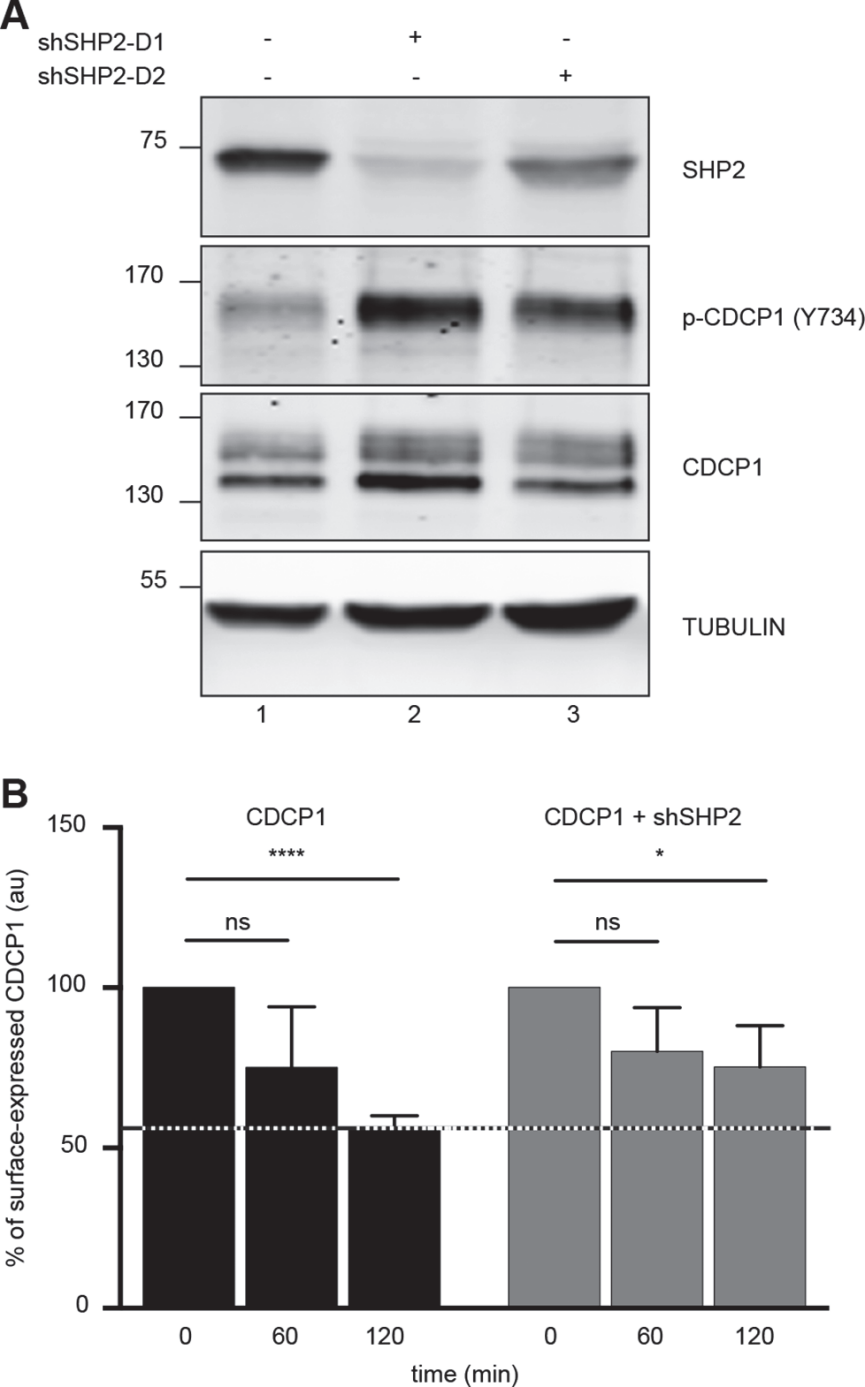
SHP2 regulates the phosphorylation and internalization of CDCP1. **A.** HeLa cells stably transfected with an empty vector or with a WT-CDCP1 construct were stably transfected with a SHP2-targeting shRNA (D1 or D2), as indicated. Total cell lysates were prepared and analyzed by western blotting with the antibodies indicated. **B.** Stable HeLa-CDCP1 and HeLa-CDCP1-shSHP2 D1 cell lines (described above and in the experimental procedures) were first incubated with an anti-CDCP1 antibody at 4°C. The cells were washed and incubated at 37°C for the times indicated, to allow internalization of the CDCP1-antibody complexes. The cells were then incubated again at 4°C with the appropriate secondary antibody, and the amount of CDCP1 remaining at the cell surface was analyzed by flow cytometry. The results are indicated as a percentage of membrane CDCP1 ± SEM for three independent experiments. ns: p > 0.05 *: p = 0.03; ****: p = 10^–4^ in non-parametric Student's *t* tests. The data shown are representative of at least three independent experiments performed in triplicate.

We used two different shRNA expression constructs to knock down SHP2 expression. HeLa-CDCP1 cells were transfected with these constructs, separately, and we used the appropriate selection agent to select for stable expression, to generate two cell populations expressing one or the other shSHP2 construct. The cells were left untreated, harvested and lysed as described. We found that shRNA-D1 knocked down SHP2 levels more effectively than the shRNA-D2 (Fig 6A, upper panel, compare lanes 2 and 3). The membranes were hybridized with a commercially available serum directed specifically against the phosphorylated Y734 residue of CDCP1 (see experimental procedures). The specific knockdown of SHP2 was accompanied by a large increase in phosphorylation of the Y734 residue of CDCP1 (Fig 6A, p-CDCP1 panel). This increase in phosphorylation was proportional to the decrease in SHP2 levels (Fig 6A, p-CDCP1 panel, compare lanes 2 and 3). Thus, SHP2 regulates the level of phosphorylation of CDCP1, although it remains unclear whether this regulation is direct or indirect.

### SHP2 is required for CDCP1 internalization

Kollmorgen *et al*. recently showed, in a study assessing the potential therapeutic value of an anti-CDCP1 antibody, that incubation with this antibody triggered the phosphorylation of CDCP1, its translocation to lipid rafts and the down regulation of CDCP1 [35]. We reproduced CDCP1 phosphorylation by incubating our different cell lines with various anti-CDCP1 antibodies (data not shown). We also detected a large decrease in the amount of CDCP1 protein after 60 and 120 minutes of incubation of HCT 116 cells with an anti-CDCP1 antibody (data not shown). We therefore investigated the possible involvement of SHP2 in this regulatory process. We evaluated the effect of antibody-mediated CDCP1 triggering, by assessing the presence of CDCP1 at the cell surface by flow cytometry at different time points. Briefly, as described in the experimental procedures section, the cells were first labeled with an anti-CDCP1 antibody, at 4°C, to prevent the internalization of the immune complexes. The cells were then returned to 37°C conditions and incubated for the indicated times. CDCP1 labeling of the cell surface was revealed by incubation with a specific Alexa 647-conjugated secondary antibody at 4°C. The cells were washed and surface fluorescence was quantified by flow cytometry. Following the incubation of HeLa-CDCP1 cells with an anti-CDCP1 antibody for 120 minutes (Fig 6B, black bars, CDCP1), we found that 50% of the CDCP1 present was taken up by the cells. This effect was weaker in cells in which SHP2 expression was down regulated by shRNA (Fig 6B, grey bars CDCP1 + shSHP2). More specifically, about 25% of the CDCP1 was internalized within 60 minutes, and this proportion remained unchanged after 120 minutes. Our results indicate not only that SHP2 regulates the level of phosphorylation of CDCP1, but also that it is involved in regulating the fate of CDCP1 at the cell surface following triggering with an anti-CDCP1 antibody.

## Discussion

We found that the intra cytoplasmic part of CDCP1 contained an ITAM-like sequence. The environment surrounding the Y734 and Y743 tyrosine residues delineates this motif. When phosphorylated, these two residues would create specific docking sites for signaling molecules bearing tandem SH2 domains, such as the cytosolic phosphatases SHP1 and SHP2. As SHP1 expression is largely restricted to the hematopoietic lineage, we investigated the possible association of SHP2 with CDCP1. We found that SHP2 bound CDCP1 directly when the Y734 and/or Y743 residues of this protein were phosphorylated. Both phosphorylated tyrosine residues were required, and this interaction was lost when both residues were replaced with phenylalanine residues. However, from our experiments, it seems that the preferential interaction site of interaction of SHP2 for CDCP1 is the phosphorylated Y734 residue, as more than 80% of the interaction is lost when the Y734 residue is mutated to a non phosphorylable residue. The cytoplasmic region of CDCP1 contains five tyrosine residues (Y707, Y734, Y743, Y762, and Y806), each of which constitutes a potential phosphorylation site. The Y734 residue has been shown to be a key element in CDCP1-mediated signaling events. Benes *et al*. have suggested that SRC family kinases first phosphorylate the Y734 residue, subsequently associating directly with this residue and strengthening the CDCP1-SFK interaction. The SFKs then phosphorylate the Y762 residue, which becomes a docking site for PKCδ [14]. It has recently been reported that the induction of CDCP1 phosphorylation triggered a set of discrete and specific signaling pathways, leading to the phosphorylation of SHP2 in HCT 116 colorectal cancer cells [15]. However, they obtained no evidence to suggest that CDCP1 and SHP2 were in the same complex. Our data suggest that this interaction is very transient or labile, but stabilized by the use of catalytically inactive “substrate-trapping” constructs. This may explain why this interaction has been overlooked in previous studies.

Altogether, we showed that SHP2 interacts with CDCP1 in response to a PerVO3 treatment or to an anti-CDCP1 antibody treatment, or to cell confluence that could correspond to a more physiological stimulus and would fit with the reports from several groups that showed that anti-CDCP1 antibodies induce the phosphorylation of CDCP1 [14,15,34]. These results were confirmed in at least three different cancer cell lines including HCT116, PC3 and SW620 (not shown) and in transfected-HeLa cells. We confirmed that culturing cells in the presence of various monoclonal antibodies directed against the extracellular part of CDCP1 induces an important phosphorylation of CDCP1 that is followed by a dramatic down modulation of CDCP1 expression [15,34]. Kollmorgen et al recently showed that this down regulation was due to an epoxomycin/chloroquine-sensitive protein-degradation process. They also showed that, in response to specific antibody triggering, CDCP1 was relocated to lipid rafts and internalized. In this study, we showed, by the shRNA-mediated knockdown of SHP2 expression, that CDCP1 phosphorylation levels were higher in the absence of SHP2. We were unable to demonstrate the direct dephosphorylation of CDCP1 by SHP2, and we therefore cannot rule out the possibility that this effect is not direct. However, we clearly showed that, in the absence of SHP2, despite the stronger phosphorylation of CDCP1, this protein was not taken up when the cells were cultured in the presence of a specific anti-CDCP1 antibody. The mechanism of CDCP1 internalization has not been studied to date, and it is unclear how it could be regulated by SHP2. Our preliminary data indicate that CDCP1 is mostly phosphorylated when expressed at the cell surface, whereas it is mostly unphosphorylated after its internalization (data not shown). This would suggest that the dephosphorylation of CDCP1 may be required for its internalization. However, the use of the specific SHP2 inhibitor NSC-87877 (Tocris Bioscience) did not provide any conclusive result and the question regarding whether CDCP1 internalization is regulated by the activity of SHP2 or by its association and potential recruitment of other molecules remains open. CDCP1 was also co-immunoprecipitated with caveolin-1 (CAV1), a major component of the caveolae (not shown). SHP2 has been shown to interact with CAV1 and to regulate Src activity [36]. It is possible that SHP2 regulates the uptake of CDCP1 by recruiting CAV1. These mechanisms are currently under investigation.

Benes *et al*. showed that CDCP1 was no longer phosphorylated by SFK when Y734 was replaced by a phenylalanine residue. We found that the CDCP1 double mutant (Y734F and Y743F, referred to as DM in Fig 6) was phosphorylated after pervanadate treatment. Thus, even though the Y734 and Y743 residues could no longer be phosphorylated, the CDCP1 protein nonetheless underwent phosphorylation in our study. The reasons for this remain unclear, but may be related to the pervanadate treatment, which may reveal mechanisms exclusively due to tyrosine phosphatase inhibition. However, this phosphorylation of the CDCP1 double mutant construct is an appropriate internal control in our experiments and the absence of interaction with SHP2 validates the specificity of our protocols.

Mutations of the SHP2 gene are encountered in 50% of patients with Noonan syndromes and 35% of those with juvenile myelo-monocytic leukemia (JMML). Activating mutations have also been shown to cause sporadic solid tumors [37]. SHP2 has been shown to mediate v-SRC-induced morphological changes and is known to be an effector of EGF-R [19]. In an elegant approach, Gusenbauer *et al*. recently showed that, in the presence of HGF, CDCP1 interacts with EphA2 and EGF-R, consistent with the notion that this protein may play an important role in receptor crosstalk [38]. Our data, showing that CDCP1 can recruit SHP2 and that this association may regulate the fate of CDCP1, may have important consequences for our understanding of this crosstalk between receptors, in which CDCP1 is involved.

SHP2 and SRC associate with the same residue (Y734) of CDCP1. These two proteins may therefore compete for binding to this residue, the molecular fate of CDCP1 and the signaling events downstream from CDCP1 depending on the association favored. An inverse correlation between the phosphorylation of CDCP1 and that of FAK phosphorylation has been reported and Wortmann *et al*. recently reported evidence of SRC switching between CDCP1 and FAK [39]. SHP2 has been reported to regulate lamellipodium formation by regulating the phosphorylation of FAK [26]. It is thus tempting to speculate that SHP2 may, by competing with Src, participate in the molecular switching described by Wortmann *et al*. SHP2 may thus be the phosphatase involved in CDCP1 dephosphorylation during cell adhesion.

Since the initial description of this protein in 2001, high levels of CDCP1 have been shown to be associated with a higher frequency of solid tumors in various models, including colon, prostate, pancreas, lung, renal and skin cancers [2,4–7]. It has been suggested that the up regulation of CDCP1 promotes resistance to anoikis, invasive behavior, dissemination and, ultimately, metastasis. CDCP1 seems to be a promising target for novel therapies, but we still know little about its mode of action, its partners, and the signaling pathways downstream of CDCP1. The function of CDCP1 in the mechanism of resistance to anoikis and the promotion of metastasis has been shown to be dependent on the activity of SRC family kinases [5]. Hara *et al*. recently showed that SHP2 acts in association with SIRP1α to regulate anoikis in c-SRC-transformed cells [40]. Higher CDCP1 levels might titrate or displace SHP2 from SIRP1α, thereby decreasing the involvement of SHP2 in the induction of anoikis.

Since its characterization, it is known that CDCP1 is expressed at the cell surface as a 130kDa protein. This protein has been shown to be cleaved by several proteases such as trypsin, matriptase and plasmin, generating a 70 kDa protein [41]. It was recently proposed that the cleaved CDCP1 would mainly occur through plasmin in vivo and would have a pro-survival function during metastatic colonization [42]. In our hands, the only cell line that showed an efficient cleavage of CDCP1 is the PC3 prostate cancer cell line and we observed an equivalent binding of SHP2 to the 130kDa or to the 70 kDa phosphorylated forms of CDCP1 (not shown). In other words, SHP2 could bind indifferently to the cleaved or non-cleaved CDCP1 proteins. This observation would be in agreement with the observation reported by Spassov et al. demonstrating that the intra cellular domain of CDCP1, that is present in the 130 kDa and the 70 kDa forms, is necessary and sufficient to inhibit adhesion of HEK 293 cells [43].

The use of specific anti-CDCP1 antibodies has been proposed as a therapeutic strategy for targeting cancer cells overexpressing CDCP1. However, the use of such strategies is limited by the current lack of knowledge about functional effects downstream of the antibody-mediated triggering of CDCP1. We have identified a new interactor of CDCP1 and we demonstrate its importance for the internalization process following the binding of a specific antibody.

The tyrosine phosphatase SHP2, is a “druggable” signaling molecule, due to its enzymatic activity, and is currently the subject of drug development studies. It has been suggested that it might be possible to inhibit cancer cell metastasis by interfering with SHP2 activity [44]. However, caution in interpretation is required, depending on the cell type and CDCP1 expression status. Indeed, our results indicate that SHP2 knock-down may increase the production and phosphorylation of CDCP1, with potentially adverse effects due to the promotion of invasion and dissemination. Thus, more detailed knowledge of the interactors of CDCP1 and their functions at the molecular or cellular level will be required to decipher more precisely the therapeutic potential of treatment strategies targeting CDCP1.
